# ICD-Based Cause of Death Statistics Fail to Provide Reliable Data for Medical Aid in Dying

**DOI:** 10.3389/ijph.2023.1606260

**Published:** 2023-08-11

**Authors:** Uwe Güth, Christoph Junker, Marion Schafroth, Shaun McMillan, Andres R. Schneeberger, Constanze Elfgen, Edouard Battegay, Rolf Weitkunat

**Affiliations:** ^1^ Department of Breast Surgery, Brust-Zentrum Zürich, Zurich, Switzerland; ^2^ Faculty of Medicine, University of Basel, Basel, Switzerland; ^3^ Federal Statistical Office, Neuchâtel, Switzerland; ^4^ EXIT, Zurich, Switzerland; ^5^ Department of Psychiatry, University of California San Diego, La Jolla, CA, United States; ^6^ Faculty of Medicine, University of Witten/Herdecke, Witten, Germany; ^7^ International Center for Multimorbidity and Complexity in Medicine (ICMC), University of Zurich, Zurich, Switzerland; ^8^ Department of Psychosomatic Medicine, University Hospital Basel, Basel, Switzerland; ^9^ Merian Iselin Clinic Basel, Basel, Switzerland

**Keywords:** medical aid in dying, physician assisted suicide, ICD classification, cause-of-death statistics, end of life decisions

## Abstract

**Objectives:** To evaluate the most recent developments of medical aid in dying (MAID) in Switzerland and to test the reliability of reporting this phenomenon in cause of death statistics.

**Methods:** By reviewing the MAID cases between 2018 and 2020, we compared the diseases and conditions underlying MAID reported by the ICD-based statistics provided by the Swiss Federal Statistical Office (FSO, *n* = 3,623) and those provided by the largest right-to-die organization EXIT (*n* = 2,680).

**Results:** EXIT reported the motivations underlying the desire for death in a mixture of disease-specific and symptom-oriented categories; the latter including, for example, multimorbidity (26% of cases), and chronic pain (8%). Symptom-oriented categories were not included in the ICD-based FSO statistics. This led to the fact that the distribution of the diseases/conditions underlying MAID differed in 30%–40% of cases between both statistics.

**Conclusion:** In order to reliably follow developments and trends in MAID, the diseases/conditions underlying the wish to die must be accurately recorded. Current methods of data collection using the ICD classification do not capture this information thoroughly (“MAID gap”). Newly created ICD codes for MAID must include both disease-specific and symptom-oriented categories.

## Introduction

Medical aid in dying (MAID) is a comparatively new societal and medical phenomenon. In recent years, most Western countries have increasingly acknowledged individual autonomy regarding controlling aspects of one’s own dying and death. This is particularly true for patients suffering from terminal disease or experiencing symptoms and functional impairments which are the source of intolerable suffering [1–7]. Consequently, a growing number of countries, including Switzerland, Belgium, the Netherlands, Luxembourg, Austria, Spain, Colombia, Canada, some US states (California, Colorado, Hawaii, Maine, Montana, New Jersey, New Mexico, Oregon, Washington state, Vermont, and the District of Columbia), New Zealand, and the six Australian states conditionally allow for different forms of planned MAID [8–10].

Causes of death are compiled in statistics according to rules set by the World Health Organization (WHO), i.e., the diseases and conditions underlying death are coded based on the International Statistical Classification of Diseases and Related Health Problems (ICD; during the study period the 10th version in use: ICD-10) [11, 12]. In all countries where any forms of assisted dying are permitted, it was soon realized that the ICD-10 classification does not provide a separate code for MAID. Hence, MAID is not adequately reflected in the national cause of death statistics. This “MAID gap” results in a lack of information about the significantly evolving phenomenon and its impact on mortality or causes of death. In consequence, politicians, lawyers, and healthcare workers cannot make adequate and reliable conclusions on the prevailing stance and reality of behaviors related to MAID [13]. There is an urgent need for valid data and recording of MAID, especially for guidance concerning controversial social and medical ethics debates, including the ability to identify abusive euthanasia practices.

When compared globally, Switzerland has a longer history of legal and documented MAID than all other countries combined, with its current societal and legal framework already in practice for about 35 years [14–17]. In the early 2000s, fewer than 100 cases occurred per year. By 2018, this number had increased to 1,176 cases, representing 1.8% of all deaths in Switzerland [14]. In Table 1, we summarize the key points of the conditions developed for the legal and medical-ethical framework of MAID, with Assisted Suicide (AS) as the only method permitted for MAID in Switzerland. In contrast, active euthanasia (killing on request) is a criminal offense, according to the Swiss Criminal Code [14–17].

**TABLE 1:** The development of the legal and medical-ethical framework conditions of assisted suicide in Switzerland (Zurich, Switzerland, 2023).

**The Criminal Code:** The Swiss model of AS was developed in the 1980s and designed with a framework that included comparatively open legal regulations in the first half of the last century [14–17]. According to Article 115 of the Swiss Criminal Code, which entered into force in 1942, AS is only illegal in cases where it is carried out “for selfish motives” [30].
In contrast, direct active euthanasia, i.e., killing on request, even if requested seriously and insistently, is a criminal offence according to the Articles 111 (“Intentional homicide”), 113 (“Manslaughter”) and 114 (“Homicide at the request of the victim”).
**The *Swiss Academy of Medical Sciences* (SAMS):** Since the Swiss legislature initially did not enact specific regulations for organized medical aid in dying (in particular a legal framework for AS), most recently in a consultation in 2011, and Swiss courts grant the medical profession a high degree of personal responsibility in the question of AS, the medical authorities had to formulate binding rules for themselves about this issue.
This social and medico-ethical development process had been adopted by the SAMS. This institution, founded in 1943, sees itself as a bridge builder between science and society. The activity of the SAMS is, among others, directed on the “clarification of ethical questions in connection with medical developments and their effects on society” [31]. As a rule, the guidelines developed by the SAMS are incorporated as an ethics protocol into the *Swiss Medical Association (Foederatio Medicorum Helveticorum,* FMH) Code of Professional Conduct and are thus binding for its members.
The SAMS guidelines for AS have changed over time from categorical rejection to a more flexible stance.
For a long time, the SAMS kept a low profile with regard to this topic. With the opinion that AS is “not a part of medical practice” because it contradicts the goals of medicine, further statements on the subject were not even considered necessary.
Then, in 2004, a revision of the “End-of-life care” guidelines was provided. Here, it was stated for the first time that a physician may, on the basis of a personal decision of conscience, assist in suicide if there is a serious disease that will lead to death in the foreseeable future [32].
During the following years, however, the right-to-die organizations in Switzerland have always gone further in assisting those who wish to die. The principle that a disease leading to death in the foreseeable future is imperative for the approval of AS was never considered binding by them. For many years, *EXIT*, which is the largest right-to die organization in the country, has propagated “old age assisted suicide” for elderly people in cases “when the sum of their pain and infirmity is perceived as an unbearable state of suffering” [33].
In 2018, the SAMS revised its recommendations on medical aid in dying (“Management of dying and death”) [34]. The key criteria for the medical profession to participate in AS was now no longer that the final phase of life, but that “symptoms of disease and/or functional impairments” are present, which are the “source of intolerable suffering.”
For more than 4 years, however, the FMH refused to adopt this updated version as a professional ethics guideline in its statutes. The representatives of the FMH criticized that the revised recommendations represent a “massive expansion of the scope of (...) permissible assisted suicide” and clearly depart from their original objective to help only terminally ill people” [35]. The core of the criticism, however, was that allowing AS for people who perceive their suffering as unbearable would mean considerable uncertainty for the physicians concerned, and that the revised guidelines would make it difficult for them to define clear limits for their participation in AS, since the term “intolerable suffering” is too vague and depends heavily on the patient’s subjective assessment. Thus, for many years, a smoldering conflict situation existed in Switzerland with regard to medical ethics and professional law.
In order to put an end to this unsatisfactory state of affairs, the SAMS and the FMH set up a joint working group in 2021, which once again revised the medical-ethical guidelines on AS. The updated version of the new recommendations includes a few specifications (e.g., in the subchapters “Capacity” and “Autonomous wishes”), but above all the recognition that well-documented “severe suffering” is sufficient as a criterion for granting AS [34]. The FMH adopted the reformulated document in May 2022; as a result, the revised guidance was incorporated as a code of ethics into the FMH Code of Professional Conduct [35].
Note: The legal framework for AS is not defined by the SAMS Guidelines, but by the Swiss Criminal Code (Strafgesetzbuch) and the Federal Narcotics and Therapeutic Products Acts. The SAMS guidelines are medical-ethical guidelines and have no immediate legal force.
**The high level of social acceptance:** The current practice of AS is widely accepted among those living in Switzerland. This was mirrored in the results of a referendum that was held in Canton of Zurich in May 2011, titled “Stop Assisted Suicide”, which was intended to put an end to the practice. The proposal was rejected by 84% of the vote [19].
**Interaction between medical and non-medical responsibilities:** In the practical application of AS a close interaction between medical and non-medical responsibilities has developed [14–17].
The non-medical responsibilities have been taken over by the staff of the various right-to-die organizations. The role of the doctor in this process consists principally in the prescription of a lethal dose of sodium pentobarbital after careful assessment of the underlying disease and the capacity of judgement of the patient. During the suicide itself as a rule a member of staff of the right-to-die organization is present but not necessarily the doctor.
After the onset of death, the AS must be reported to the authorities as an “extraordinary death.”

AS, Assisted suicide; FMH, Swiss Medical Association (Foederatio Medicorum Helveticorum); SAMS, Swiss Academy of Medical Sciences.

In this study, we have evaluated the most recent developments of AS in Switzerland (2018–2020) with a special focus on cases without a clearly defined disease leading to death in the foreseeable future. Using AS resulting from age as an example, we depict AS as not being properly and comprehensively represented in ICD-based cause of death statistics and recommend how this could be achieved in the future with a symptom-based, easy-to-handle extension of the classification code.

## Methods

For this study, we analyzed all death cases in Switzerland from 2018 to 2020; during these 3 years, 211,063 people died (Table 1). Typical for an aging Western population, the median age at death was high, at 83 years (men: 80 years, women: 86 years).

In 2020, the first COVID-19 pandemic year, excess mortality was observed. While there were usually around 70,000 deaths in Switzerland in a normal year, in 2020 there were 76,195 deaths which signified an increase of 12.4% compared with the previous year. In 2020, 9,331 people residing in Switzerland died with COVID-19 as the primary cause of death. The proportion of deaths with COVID-19 as the main cause of death was 12.2%, third only to cardiovascular disease (26.9%) and cancer (22.2%) [18].

**The data source for recording AS cases including underlying diseases and conditions:** In all AS cases in Switzerland, it is mandatory that the physician who prescribes the lethal medication reports the motives of those who wish to die in detail and confirms in writing that the patient was of sound judgment regarding their wish to end their life. This medical report, which includes information on the disease or the condition underlying the AS, is owned by the right-to-die organizations. In general, a member of these organizations is present during the suicidal act. Immediately after the patient’s death, a forensic medical doctor or a public health officer, who investigates and certifies the circumstances of the death, receives a copy of this report. Based on this report, the cause of death and the disease underlying the AS are then documented on the death certificate; this information is then transferred to the Federal Statistical Office (FSO) which is responsible for the Swiss cause of death statistics.

The medical reports are archived by the right-to-die organizations and the involved institutes of forensic medicine. The reports are not available to the FSO.

**Reporting AS in Switzerland:** All AS cases are recorded in the Swiss cause of death statistics provided by the FSO. The annual case numbers as well as information regarding sex and age at the time of death (younger vs. older than 65 years) are regularly published on the FSO website. In-depth analyses, which also report the diseases underlying assisted suicides, are not published at regular intervals. The last publication is dated 2016, which summarizes the cases until 2014 [12].

Since the mid-1990s, the FSO has received isolated notifications of AS. Since the ICD-10 classification does not provide a separate code for MAID, the FSO created its own code for AS in 2009, applicable only in Switzerland. The cases are logged with code X61.8 as the direct cause of death, i.e., the FSO has given the established code X61 which is used for “Intentional self-poisoning by and exposure to antiepileptic, sedative-hypnotic, antiparkinsonism and psychotropic drugs, not elsewhere classified” its own supplementary digit: X61.8 [12]. In all AS cases, the disease leading to the suffering is coded as the underlying cause of death. Subsequently, the FSO interprets AS as “the last resort taken at the end of a serious disease” [12].

The FSO cause of death statistics refers only to persons who are legal residents of Switzerland, i.e., the permanent resident population regardless of nationality and place of death. This implies that citizens of other countries who use the AS option in Switzerland (“suicide tourism”) are not included in these statistics. The cause of death statistics data analyses and publications are governed by the Swiss Civil Code and the Federal Statistical Act and are carried out according to the Swiss Federal Act on Data Protection (SR 235.1) [19].

**Two independent Swiss associations were founded in 1982 under the name *EXIT*:** *EXIT,* the section for German-speaking Switzerland and the Italian-speaking canton of Ticino, and *EXIT A.D.M.D.* in the French-speaking areas of Switzerland. These two sister organizations are the largest of six right-to-die organizations in Switzerland. Combined they currently have 185,000 members. Their main objective is “to advocate for personal responsibility and self-determination in the last phase of life and for people who suffer from severe ailments” [20]. *EXIT* issues “living wills, palliative care through own foundation, counselling in case of illness, old-age issues, suicide prevention, and dignified end-of-life care” [21]. In Switzerland, *EXIT* plays an important role in the public perception of AS and explicitly advocates the right to AS. *EXIT* offers its services exclusively to Swiss citizens and legal residents in Switzerland.

*EXIT* has assisted in suicides and has been publishing the number of annual cases since 1987. As of 2010, they have also been reporting diseases or conditions underlying the wish to die [22].

## Results

Between 2018 and 2020, the FSO registered a total of 3,623 cases of AS (Table 2). This corresponds to 1.7% of all death cases. During our 3-year observation period, the annual number of AS rose slightly (2018, *n* = 1,176; 2019, *n* = 1,196, compared with the previous year: +1.7%; 2020, *n* = 1,251, +4.6%; Table 2). Due to the COVID-19 pandemic and its excess mortality, for the first time since AS recordings began in Switzerland 23 years ago, there was a slight decrease in the share of AS cases in the total number of deaths compared to the previous year (2018 and 2019: 1.8% vs. 2020: 1.6%). AS was generally chosen by older people (median age: 80 years) with a predominance of women (58.8%; chi-square statistic = 83.3, *p* < .001).

**TABLE 2:** Death cases in Switzerland (2018–2020) with particular consideration of assisted suicide as the cause of death (Zurich, Switzerland, 2023).

Time period	Entire period 2018–2020	2018	2019	2020
**All death cases (men and women)**	211,063	67,088	67,780	76,195
Median age at death	83	83	83	84
Cause of death: AS (% of all cases)				
Percentage increase compared with the previous period	3,623 (1.7)	1,176 (1.8)	1,196 (1.8)+1.7%	1,251 (1.6)+4.6%
Median age at death	80	80	80	81
**Men**				
All male death cases	102,778	32,398	32,756	37,624
Median age at death	80	80	80	81
Cause of death: AS (% of all cases)	1,492 (1.5)	499 (1.5)	483 (1.5)	510 (1.4)
Median age at death	80	80	80	80
**Women**				
All female death cases	108,285	34,690	35,024	38,571
Median age at death	86	86	86	86
Cause of death: AS (% of all cases)	2,131 (2.0)	677 (2.0)	713 (2.0)	741 (1.9)
Median age at death	81	81	81	82

AS, assisted suicide.

During our 3-year study period, *EXIT* facilitated and accompanied AS in 2,680 people (2018, *n* = 905; 2019, *n* = 862; 2020, *n* = 913), which accounts for 74.0% of all AS cases registered in Switzerland.

**FIGURE 1:**
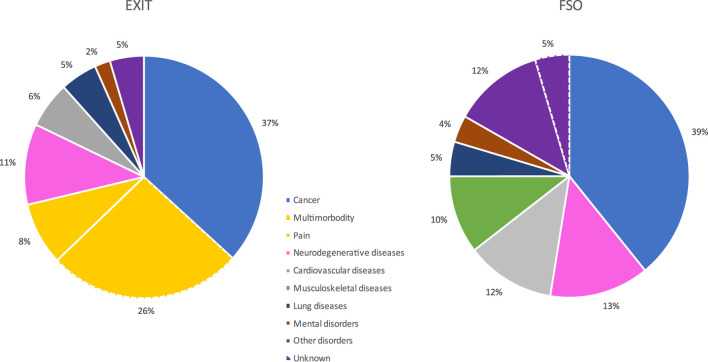
Assisted suicide in Switzerland and the distribution of its underlying diseases and conditions (rounded percentages): Comparison between the ICD-based cause of death statistics used by the Swiss Federal Statistical Office (FSO) and a more symptom-based classification system used by *EXIT* (Zurich, Switzerland, 2023).

Table 3 and Figure 1 describe the diseases and conditions which were recorded as the underlying disease which led to AS. The reporting of data regarding underlying diseases and conditions is consistent with that of the 2016 FSO report on AS [12]. According to the FSO cause of death statistics, the most common underlying condition for AS in Switzerland was cancer (ICD-10 code C00-C97; *n* = 1,415; 39.1%, range 38.3%–39.8%). This represents 2.8% of all cancer-related deaths (3.2% of all female and 2.4% of all male cancer-related deaths). Cancer was more likely to be the most underlying condition in men compared to women (44.6% vs. 35.2%; chi-square = 32.4, *p* < .001). Individuals who choose AS because of malignant diseases were considerably younger than those who chose AS in connection with other diseases (median ages at death: 75 years vs. 84 years). The *EXIT* statistics also show cancer as the most common disease coded. The proportion of cancer-related AS cases shows similar values in both statistics (37% vs. 39%).

**TABLE 3:** Assisted suicide cases in Switzerland (2018–2020): distribution of underlying diseases reported by EXIT and the Swiss Federal Statistical Office (FSO) Cause of Death Statistics (Zurich, Switzerland, 2023).

	EXIT	FSO
**All cases with AS**	2,668	3,623
Median age of death (range)	81 (20–103)	80 (20–104)
**Underlying disease reported:**		
**1. Cancer (%)**	979 (36.7)	1,415 (39.1)
Median age at death (range)	75 (20–99)	75 (20–99)
**2. Other diseases (%)**	1,689 (63.3)	2,040 (56.3)
Median age of death (range)	84 (29–103)	83 (27–104)
**2.1. Multimorbidity**	698 (26.2)	not reported
Median age of death (range)	89 (60–103)	
**2.2. Neurodegenerative diseases**	287 (10.8)	487 (13.4)
Median age at death (range)	76 (36–98)	76 (27–103)
2.1.1 Parkinson’s disease	89 (3.3)	105 (2.9)
2.1.2 Amyotrophic lateral sclerosis (ALS)	63 (2.4)	83 (2.3)
2.1.3 Multiple Sclerosis (MS)	49 (1.8)	55 (1.5)
2.1.4 Dementia	58 (2.2)	63 (1.7)
2.1.5 Eye disease	28 (1.0)	not reported
2.1.5 Other neurodegenerative diseases	not reported	181 (5.0)
**2.3. Chronic pain**	225 (8.4)	not reported
Median age of death (range)	82 (29–100)	
**2.4 Cardiovascular diseases**	165 (6.2)	434 (12.0)
Median age at death (range)	84 (53–101)	87 (47–103)
2.4.1 Heart disease	81 (3.0)	not reported
2.4.2 Stroke	84 (3.2)	not reported
**2.5 Musculoskeletal diseases**	not reported	376 (10.4)
Median age at death (5th to 95th percentile)		86 (39–100)
**2.6 Lung diseases**	134 (5.0)	168 (4.6)
Median age at death (range)	77 (54–97)	77 (54–100)
**2.7 Mental disorders**	56 (2.1)	132 (3.6)
Median age at death (range)	67 (35–88)	81 (29–99)
**2.8 Other disorders**	124 (4.6)^a^	443 (12.2)
Median age at death (range)	75 (35–89)	86 (47–104)
**3. Unknown**	In all cases an underlying condition had been reported	168 (4.6)
Median age at death (5th to 95th percentile)		85 (32–101)

*EXIT*: In addition to the categories “multimorbidity” and “chronic pain,” there are further symptom-based categories: “eye disease” (*n* = 28, 1.0% of all AS cases), “peripheral neuropathy” (*n* = 25, 0.9% of all AS cases) and quadriplegia (*n* = 10, 0.4% of all AS cases) are only listed in the *EXIT* statistics but not in the ICD-based FSO cause of death statistics.

^a^ *EXIT*, “other disorders” includes: peripheral neuropathy (*n* = 25), renal disease (*n* = 16), quadriplegia (*n* = 10), and HIV (*n* = 7). These conditions are reported in the annual EXIT reports and account for 2.2% of AS cases. We omit to report these diseases/conditions separately because they are relatively rare and each account for <1% of cases.

However, there was a substantial difference in the recording and reporting of the underlying non-cancer-related diseases and conditions of AS between the FSO’s cause of death statistics and *EXIT*’s published data for the cases where members of their staff accompanied those willing to die. The FSO’s death register aligns AS cases exclusively with the international guidelines from the ICD-10 classification. *EXIT* also reported approximately 65% of their AS cases along these lines. However, in more than one-third of the cases, *EXIT* detached itself from the restrictive guidelines of the ICD classification and did not report any specific underlying disease. In these cases, a multitude of symptoms leading to the desire for death was reported. This results in clear differences between FSO- and *EXIT*-reported distribution of the underlying diseases (Table 3; Figure 1). In *EXIT*’s recording of the underlying diseases and conditions of AS, “multimorbidity” is the second most common cause of the wish to die, accounting for 26.2% of AS cases. Furthermore, the category “chronic pain,” with 8.4% of all AS cases the fourth most frequent category, is also exclusively listed in the *EXIT* statistics but not in the ICD-based FSO cause of death statistics. In Figure 1, these symptom-based categories, which combined account for 34.6% of all AS cases accompanied by *EXIT*, are particularly highlighted with yellow slices in the diagram.

## Discussion

As we critically examined the results, it became surprisingly clear to see the distribution of diseases and conditions underlying AS differ in at least one-third of cases between the FSO and EXIT data. After all, both statistics stem from the same source, namely the report of the physician who made the AS possible by prescribing the lethal drug. On closer examination, however, this raises one main question: why do ICD-based cause of death statistics only apply in a limited capacity for AS? This might mainly be due to the ICD classification not providing a code for MAID. But even if a national organization provides an extension to an existing code, as the Swiss FSO has been doing for many years (using the “suicide code” X61.8 for AS cases), for many of these cases even this does not map the causal chain accurately. The WHO rules stipulate that the cause of death should be the disease that is the root cause leading to death. In this regard, AS is usually the last resort taken at the conclusion of a serious disease [23]. As long as the main criterion is observed that only patients suffering from a severe illness that will lead to death in the foreseeable future can choose the option of AS (terminal illness requirement), the ICD system is also capable of representatively describing these cases.

However, in countries such as Switzerland, the Benelux countries, Spain, Austria, Colombia, and Canada, the regulations for MAID go beyond the terminal illness requirement and also accept symptom-based conditions which implies a state of intolerable suffering as an indication for assisted dying. It is precisely this symptom-oriented and patient-related subjective extension of inclusion criteria for MAID that pushes the ICD classification to its limits. In cases of chronic pain syndromes such as severe rheumatic diseases and polyneuropathy or in diseases that lead to severe limitations of mobility such as Parkinson’s disease, multiple sclerosis, stroke-related paralysis, and traumatic spinal cord injuries, the ICD classification might be able to depict the motivational factors of the person willing to die. The above-mentioned diseases usually represent severe, immobilizing, and invalidating chronic conditions. For the patients concerned, this means a high level of suffering, usually without any hope of an improvement in their situation. Yet, according to the official definition listed as the cause of death on the death certificate, these diseases are not direct causes of death.

Unfortunately, the ICD classification system completely fails in the case of AS due to age-related complaints and conditions where the wish to die refers to a variety of influencing factors such as limited mobility, reduced vision, and hearing, as well as social factors such as institutionalization with a loss of autonomy and control or loneliness after the passing of family or friends. There is no code to express a life situation in which future prospects are exclusively composed of worsening complaints, limitations, and dependencies already present that are not going to improve.

*EXIT* has chosen a different approach in the statistical presentation of those AS cases accompanied by its staff members. The motivations underlying the desire for death are represented in a mixture of disease-specific and symptom-oriented categories. The “classic” disease-specific, and thus ICD-compatible category involves cancer. In these cases, the distinct classification practices show largely consistent figures (FSO: 39% of all AS cases vs. *EXIT*: 37%). However, if the wish to die was not related to tangible illnesses leading to a natural cause of death in the foreseeable future, but rather to symptom-based complaints, these cases were grouped separately. Consequently, “Multimorbidity” accounted for more than a quarter of *EXIT’*s AS cases; “chronic pain” was the fourth most frequent category, accounting for 8% of all cases. The median age of people for whom “Multimorbidity” was the reason for AS was 89 years. Undoubtedly, an accumulation of old age complaints was present in a majority of these cases.

The ICD classification used by the FSO cannot represent the aspects of dying for very old, frail persons, as it only includes the code R54 (“Age-related physical debility”) as a separate code. Accordingly, the “accumulation of different senile complaints” underlying old age AS cannot be properly recorded. In these cases, other diseases have to be used as “proxies” for the primary reason for AS. In elderly multimorbid patients, the selection of potential diseases which are listed in the ICD classification is usually easy to find. An analysis of the FSO data regarding the median ages of the persons who chose AS due to non-cancer-related AS categories makes it clear that a considerable number of these cases are likely to be attributable to old age AS and “proxies” were used to record these cases, e.g., cardiovascular diseases, 12.0% of all AS cases, median age at death: 87 years; musculoskeletal diseases, 10.4%, 86 years; other diseases, 12.2%, 86 years.

The comparison of the two classification systems shows that stand-alone ICD-based cause of death statistics cannot provide a clearly comprehensible coding of the current practice of AS. Classification in “proxy” categories tends to lead to a ciphering that obscures the accuracy. This could be tolerated if these “miscategorizations” pertain to only a few individual cases in MAID practice; however, it is applicable to approximately 30%–40% of all cases.

At first glance, pointing out that cause of death statistics in Switzerland do not fully reflect less than 1% of deaths might be erroneously considered irrelevant. However, the cases “lost” are the subject of one of the most relevant and recent medical-ethical debates: how does a society in which MAID has been accepted for many years, and is even regarded by many as part of the country’s national identity, handle patients who ask for assisted dying despite they are not life-threateningly ill with impending death (see Table 1)? Alternatively formulated, how do health professionals involved in MAID deal, in the absence of clear legal guidelines, with those who do not have to die but want to die?

In Switzerland, there is broad social acceptance of assisted dying [14–17, 24]. Nevertheless, critical voices worry about MAID as a “globally unique uncontrolled business model” [25]: they criticize that the right-to-die organizations, acting as associations, do not require any official authorization or license to participate in the “death market” and are alarmed about the potential danger of abusive practices. Critical voices were also raised among supporters of MAID. They fear that after its legalization, safeguards put in place for these practices might eventually be deliberately bypassed. From here, broadening the indications for AS might lead to an uncontrolled extension to persons who are not terminally ill or do not suffer from severe symptoms (*slippery slope argument*) [26]. In consequence, guidelines that clearly and bindingly regulate access to MAID have to be developed in order to protect healthy persons with a wish to die from themselves

For the advocates that believe in the principle that MAID should be used only in cases in which patients suffer from a terminal illness and/or experience unbearable and uncontrollable pain, the legalization of “old age assisted dying” goes a step too far. They suggest that the increasing prevalence of MAID could give rise to a new societal norm where the right to die implies a moral responsibility to also use this freedom. In such a “culture of euthanasia,” elderly people in need of care might feel pressured to assume their personal responsibility as a liability to those around them and relieve their relatives and society of the burden of their existence. In an extreme scenario, the ideal of a humane and self-determined ending of life free of undue suffering could evolve into a “duty to die” [27–29]. A first step towards the goal to quantify and monitor the development of this important social phenomenon would be to improve data collection methods to record MAID cases.

Thus, we strongly suggest revising the current ICD classification to incorporate codes for MAID. According to the legal regulations of countries that also accept cases beyond the “terminal illness requirement” for MAID, some new codes should be added to also include symptom-based categories. Our data analysis demonstrates that including such a change, similar to how *EXIT* summarizes its cases in its annual reports, does more justice to the precise recording of MAID than the methods currently used by the FSO in the ICD-based cause of death statistics.

In Table 4, we present a potential concept with additional codings to the existing ICD classification. Even with this modernized coding, it may be difficult to make a clear assignment to one of the categories in individual cases. Nevertheless, using the six additional newly created codes listed in Table 4 in combination with the disease-specific ICD code may allow us to specifically and easily record MAID.

**TABLE 4:** Proposal for an extended ICD-11 classification in cases with medical aid in dying including symptom-oriented categories (Zurich, Switzerland, 2023).

For our proposal, we suggest an extension of the classification from chapter 23 (“External causes of morbidity and mortality”) with a new code that exclusively describes the phenomenon of “Medical aid in dying.”		
Alternatively, “Medical aid in dying” could also be included in chapter 25 (“Codes for special purposes”).		
Subheading: Medical aid in dying PM1: Assisted suicide. PM2: Voluntary active euthanasia.		
Note: Medical aid in dying is considered as the circumstance in which a doctor prescribes a patient wishing to die a lethal substance or makes that substance available with the object of enabling the patient to die. In assisted suicide, the physical control of administering the drug is in the hands of the patient, i.e., the patients wishing to die must themselves carry out the last, decisive act of the procedure that will cause death. In contrast, in active euthanasia, the physician or healthcare professionals also administers the lethal drug.		
The underlying disease or condition recorded with the ICD-11 coding was the main reason for the wish to die.		
If the wish to die was not based on tangible illnesses leading to a natural death in the foreseeable future, but rather on other diseases or symptom-oriented complaints, additional codes (PM1.1-4; PM2.1-4) can be used.		
PM1.0 / PM2.0	The underlying ICD-coded disease was the main reason for the wish to die.	
	Comment: This code includes, for example, the most common group of indications for MAID today, namely cancer; e.g., the case of a woman who decides to have AS at a late stage of breast cancer will be coded PM1.0 or PM2.0 and 2C61 (Invasive carcinoma of the breast).	
PM1.1 / PM2.1	Medical aid in dying due to multimorbidity.	
	Comment: Multimorbidity also includes the accumulation of old age complaints. In this case, it does not matter which of the usually several existing age-related diseases and conditions is additionally coded as underlying disease.	
PM1.2 / PM2.2	Medical aid in dying due to severe neurologic-related conditions.	
	Comment: In this case, the underlying neurologic disease that was critical to the desire to die must be coded; e.g., for Parkinson’s disease: PM1.0 or PM2.0 and 8A00.0.	
	This code includes cases in which neurodegenerative diseases (e.g., Parkinson’s disease, amyotrophic lateral sclerosis, multiple sclerosis) were the main reason for the desire for death. Cases in which dementia was the underlying condition of the desire for death are excluded (→ PM1.4 or PM2.4).	
	Cases in which severe neurological limitations such as blindness and paralysis (tetraplegia) led to MAID are also included.	
	Similarly, this category also includes patients for whom the consequences of a stroke were the determining factor to choose MAID. In a symptom-based system, it makes much more sense to remove these cases from the category of “cardiovascular diseases,” in which they are currently still classified. When the consequences of a stroke lead to MAID, there is virtually always a clinical picture of severe and irreversible immobility and paralysis. This clinical picture then corresponds far more to those of the other neurologically related diseases summarized in this chapter than to those of other cardiovascular diseases, e.g., heart failure.	
PM1.3 / PM2.3	Medical aid in dying due to chronic pain.	
PM1.4 / PM2.4	Medical aid in dying due to mental disorders or dementia.	
PM1.Z / PM2.Z	Medical aid in dying, unspecified.	

MAID, medical aid in dying.

### Conclusion

MAID is a comparatively new medical and cultural phenomenon and is subject to extensive and controversial social, medical ethical, and legal discussions. In order to reliably follow developments and trends in MAID, in particular for old age assisted dying, the diseases and conditions underlying the wish to die must be accurately recorded. Current methods of data collection using the WHO ICD classification do not capture this information thoroughly today (“MAID gap”). It is therefore of the utmost importance in the near future that the WHO build the case to adequately record the phenomenon of MAID in the cause of death statistics. A newly created ICD code for MAID must include both disease-specific and symptom-oriented categories.

The “MAID gap” in the ICD classification currently affects only those few countries where MAID is legally practiced and permitted. However, it must be expected that in the coming years, many more countries will broaden their legislation about AS. Thus, regulation of the recording of MAID in the ICD classification would not only benefit a small subset of countries where the practice is already in place but will become an essential data reporting condition on a much larger, global scale.

## References

[1] European Values Study. Justfiable: Euthanasia (2022). Available from: https://www.atlasofeuropeanvalues.eu/maptool.html (Accessed March 12, 2023).

[2] Cohen J Marcoux I Bilsen J Deboosere P van der Wal G Deliens L. Trends in Acceptance of Euthanasia Among the General Public in 12 European Countries (1981-1999). Eur J Public Health (2006) 16:663–9. 10.1093/eurpub/ckl042 16641157

[3] Cohen J Van Landeghem P Carpentier N Deliens L. Public Acceptance of Euthanasia in Europe: A Survey Study in 47 Countries. Int J Public Health (2014) 59:143–56. 10.1007/s00038-013-0461-6 23558505

[4] Emanuel EJ Onwuteaka-Philipsen BD Urwin JW Cohen J. Attitudes and Practices of Euthanasia and Physician-Assisted Suicide in the United States, Canada, and Europe. JAMA (2016) 316:79–90. 10.1001/jama.2016.8499 27380345

[5] O'Neill C Feenan D Hughes C McAlister DA. Attitudes to Physician and Family Assisted Suicide: Results From a Study of Public Attitudes in Britain. J Med Ethics (2002) 28:52. 10.1136/jme.28.1.52 11834762 PMC1733504

[6] Brauer S Bolliger C Strub JD. Swiss Physicians' Attitudes to Assisted Suicide: A Qualitative and Quantitative Empirical Study. Swiss Med Wkly (2015) 145:w14142. 10.4414/smw.2015.14142 25999298

[7] Brenan M. Americans’ Strong Support for Euthanasia Persists. Washington, DC: Gallup (2018). Available from: https://news.gallup.com/poll/235145/americans-strong-support-euthanasia-persists.aspx (Accessed March 12, 2023).

[8] Wikipedia. Assisted Suicide (2023). Available from: https://en.wikipedia.org/wiki/Assisted_suicide (Accessed March 12, 2023).

[9] Mroz S Dierickx S Deliens L Cohen J Chambaere K. Assisted Dying Around the World: A Status Quaestionis. Ann Palliat Med (2021) 10(3):3540–53. 10.21037/apm-20-637 32921084

[10] Irish Hospice Foundation Paper. The International Experience of Assisted Dying (2021). Published October 2021. Available from: https://hospicefoundation.ie/wp-content/uploads/2021/10/International-Experience-of-Assisted-Dying-Oct-2021.pdf (Accessed March 12, 2023).

[11] Federal Statistical Office (FSO). Cause of Death Statistics. Death and its Main Causes in Switzerland, 2018. Neuchatel: Federal Statistical Office (2021). Published March 2021. Available from: https://www.bfs.admin.ch/bfs/en/home/statistics/health/state-health/mortality-causes-death.assetdetail.16644533.html (Accessed March 12, 2023).

[12] Federal Statistical Office (FSO). Cause of Death Statistics. Assisted Suicide and Suicide in Switzerland. Neuchatel: Federal Statistical Office (2016). Available from: https://www.bfs.admin.ch/bfsstatic/dam/assets/3902308/master (Accessed December 08, 2022).

[13] World Health Organization (WHO). Classification of Diseases/Cause of Death. Geneva: WHO (2019). Available from: https://www.who.int/standards/classifications/classification-of-diseases/cause-of-death (Accessed March 12, 2023).International

[14] Montagna G Junker C Elfgen C Schneeberger AR Güth U. Long-Term Development of Assisted Suicide in Switzerland: Analysis of a 20-Year Experience. Swiss Med Wkly (2023) 153:40010. 10.57187/smw.2023.40010 36971666

[15] Burkhardt S La Harpe R. Debates About Assisted Suicide in Switzerland. Am J Forensic Med Pathol (2012) 33:410–3. 10.1097/PAF.0b013e318273b83f 23099546

[16] Bartsch C Landolt K Ristic A Reisch T Ajdacic-Gross V. Assisted Suicide in Switzerland - An Analysis of Death Records From Swiss Institutes of Forensic Medicine. Dtsch Arztebl Int (2019) 116:545–52. 10.3238/arztebl.2019.0545 31554543 PMC6794705

[17] Schmidlin S Elger BS McLennan S. Assisted Suicide in Switzerland: Where Have We Come From and Where Are We Going? Eur J Palliat Care (2014) 21:61–5.

[18] Federal Statistical Office (FSO). Cause of Death Statistics 2020. COVID-19 Was the Third Most Common Cause of Death in Switzerland in 2020. Neuchatel: Federal Statistical Office (2022). Published August 2022. Available from: https://www.admin.ch/gov/en/start/documentation/media-releases.msg-id-90087.html (Accessed March 12, 2023).

[19] The Federal Assembly of the Swiss Confederation. Federal Act on Data Protection. Current Version; in Force: July 1, 1993; Abrogation Date: September 1, 2023 (1992). Available from: https://www.fedlex.admin.ch/eli/cc/1993/1945_1945_1945/en (Accessed March 12, 2023).

[20] EXIT-Deutsche Schweiz. What Does Physician Assisted Suicide Mean? (2019). Available from: https://exit.ch/en/englisch/faq/ (Accessed March 12, 2023).

[21] EXIT-Deutsche Schweiz. EXIT at a Glance/Who Is EXIT? (2019). Available from: https://www.exit.ch/en/ (Accessed March 12, 2023).

[22] EXIT-Deutsche Schweiz. Jahresberichte 2015-2020. Available from: https://www.exit.ch/ (Accessed March 12, 2023).

[23] World Health Organization (WHO). International Statistical Classification of Diseases and Related Health Problems. Tenth Revision, Volume 2. 4. Rules and Guidelines for Mortality and Morbidity Coding; 4.1.2 Underlying Cause of Death (2004). Available from: https://apps.who.int›handle›9241546530_eng (Accessed March 12, 2023).

[24] Canton of Zurich. Wahlen & Abstimmungen (2011). Published May 2011. Available from: https://wahlen-abstimmungen.zh.ch/internet/justiz_inneres/wahlen-abstimmungen/de/abstimmungen/abstimmungsarchiv.html?tag=15.05.2011 (Accessed March 12, 2023).

[25] Ackeret M. Die Sterbehilfe in der Schweiz Ist Längst Ausser Kontrolle (2019). Published January 2019. Available from: https://www.swissinfo.ch/ger/standpunkt_die-sterbehilfe-in-der-schweiz-ist-laengst-ausser-kontrolle/44599878 (Accessed March 12, 2023).

[26] Potter J. The Psychological Slippery Slope From Physician-Assisted Death to Active Euthanasia: A Paragon of Fallacious Reasoning. Med Health Care Philos (2019) 22:239–44. 10.1007/s11019-018-9864-8 30145689

[27] Jecker NS. Against a Duty to die. Virtual Mentor (2014) 16:390–4. 10.1001/virtualmentor.2014.16.05.oped1-1405 24847711

[28] Hardwig J. Is There a Duty Do die? Hastings Cent Rep (1997) 27:34–42. 10.2307/3527626 9131351

[29] Kious B. Burdening Others. Hastings Cent Rep (2022) 52:15–23. 10.1002/hast.1417 36226883

[30] The Federal Assembly of the Swiss Confederation. Swiss Criminal Code (1937). Decision: December 21 1937; in force: January 1 1942. Available from: https://www.admin.ch/opc/en/classified-compilation/19370083/index.html#id-ni1-ni2 (Accessed March 12, 2023).

[31] Swiss Academy of Medical Sciences (SAMW). Portrait (1943). Available from: https://www.samw.ch/en/Portrait.html (Accessed March 12, 2023).

[32] Swiss Academy of Medical Sciences. End of Life Care (2004). Published November 2004; revised in January 2013. Available from: https://www.samw.ch/dam/jcr:de64e102-1495-4c48-9fbd-1c7d4d45932f/guidelines_sams_end_of_life_2012.pdf (Accessed March 12, 2023).

[33] EXIT-Deutsche Schweiz. Voraussetzungen Einer Freitodbegleitung mit EXIT (2017). Available from: https://www.exit.ch/freitodbegleitung/voraussetzungen-einer-freitodbegleitung/ (Accessed March 12, 2023).

[34] Swiss Academy of Medical Sciences. Management of Dying and Death (2018). Published June 2018; revised in May 2022. Available from: https://www.samw.ch/en/Publications/Medical-ethical-Guidelines.html (Accessed March 12, 2023). 10.4414/smw.2018.14664 30499582

[35] FMH-Swiss Medical Association. Ärztekammer verabschiedet SAMW-Richtlinien zu «Sterben und Tod» (2022). Available from: https://www.fmh.ch/_service/aktuelles.cfm#indexes=attachment&types=3022&year=2022&class_id=154&size=30 (Accessed March 12, 2023).

